# Efficacy and safety of radiotherapy combined with immunotherapy and targeted therapy versus immunotherapy plus targeted therapy alone in unresectable hepatocellular carcinoma: a retrospective study

**DOI:** 10.3389/fonc.2025.1643304

**Published:** 2025-08-20

**Authors:** Yanling Yuan, Yongsheng Chen, Chumin Huang, Mindong Liu, Lihua Tong, Wubing Tang, Wen Yang

**Affiliations:** ^1^ Department of Oncology, The Sixth Affiliated Hospital, School of Medicine, South China University of Technology, Foshan, Guangdong, China; ^2^ Department of Radiology, The Sixth Affiliated Hospital, School of Medicine, South China University of Technology, Foshan, Guangdong, China

**Keywords:** hepatocellular carcinoma, immunotherapy, radiotherapy, targeted therapy, combination therapy, survival outcomes, toxicity profile

## Abstract

**Purpose:**

To evaluate the efficacy and safety of radiotherapy combined with immunotherapy and targeted therapy (RT+IO+T) versus immunotherapy plus targeted therapy alone (IO+T) in patients with unresectable hepatocellular carcinoma (HCC). Given the limited prospective evidence supporting the integration of radiotherapy into systemic regimens, particularly in real-world populations with advanced disease, this study aims to clarify the clinical value of this multimodal approach.

**Methods:**

This retrospective study analyzed 71 patients with unresectable HCC treated between 2020 and 2025. Patients received either IO+T (n=42) or RT+IO+T (n=29), including immune checkpoint inhibitors (ICIs) (e.g., camrelizumab), targeted agents (e.g., lenvatinib), and RT. Outcomes were assessed using the modified Response Evaluation Criteria in Solid Tumors (mRECIST) criteria. Survival analysis was performed using Kaplan–Meier and Cox regression models.

**Results:**

Compared with the IO+T group, the RT+IO+T group demonstrated superior short-term efficacy, as indicated by the objective response (69.0% vs. 35.7%, p=0.006) and disease control (89.7% vs. 57.1%, p=0.003) rates. Additionally, the median progression-free survival (PFS) and overall survival (OS) were significantly prolonged in the RT+IO+T group compared with the IO+T group (PFS: 12.6 vs. 4.6 months, p<0.001; OS: 17.8 vs. 10.9 months, p=0.009). Subgroup analyses confirmed consistent survival benefits across patient characteristics. However, the RT+IO+T group showed increased hematologic toxicity (grade ≥3 lymphopenia: 62.1% vs. 19.0%, p<0.001) and hepatic enzyme elevation (aspartate aminotransferase: 75.9% vs. 35.7%, p<0.001).

**Conclusion:**

Adding RT to IO+T significantly improved tumor response and survival in unresectable HCC, despite higher manageable hematologic and hepatic toxicities.

**Clinical significance:**

The results of this study support RT+IO+T as a promising strategy for advanced HCC, particularly in patients with high tumor burden or portal vein invasion. The synergistic effect of RT, immunotherapy, and target therapy highlights its potential to redefine treatment paradigms, although toxicity monitoring remains critical.

## 1 Introduction

Liver cancer, predominantly hepatocellular carcinoma (HCC), is the third most common cause of cancer-related death worldwide (1). In China, HCC accounts for approximately 75–85% of primary liver cancers, making it the fourth most frequently diagnosed malignancy and the second leading cause of cancer-related deaths (2). Most advanced cases are unsuitable for surgical resection and rely on non-surgical local or systemic therapies. Despite advances in early detection and treatment, a significant proportion of patients present with advanced-stage disease at diagnosis, where curative options are limited. Systemic therapies, particularly combinations of immune checkpoint inhibitors (ICIs) and targeted agents, have become the cornerstone of treatment for advanced HCC (3).

Improvements in techniques such as intensity-modulated radiotherapy (IMRT) and volumetric-modulated arc therapy have increased the use of RT for treating tumors in HCC (4). In patients with HCC complicated by venous tumor thrombus, who traditionally have a poor prognosis, with a median untreated survival of only 3–4 months, RT significantly improved both survival and quality of life (5–7).

Combining RT with immunotherapy and/or targeted therapy can have synergistic effects in treating unresectable or metastatic HCC (4, 8–10). RT enhances the efficacy of immunotherapy by triggering local inflammation and releasing tumor antigens, which boost immune responses. This combination can also induce an “abscopal effect,” in which tumor shrinkage is observed even in non-irradiated areas (11). Additionally, targeted therapy improves tumor vascular normalization, enhancing radiation sensitivity while promoting RT-induced vascular damage and apoptosis through the ceramide pathway (10, 12, 13).

However, the optimal combination of these three modalities in clinical practice remains unclear. Therefore, the present study evaluated the efficacy and safety of RT combined with immunotherapy and target therapy in patients with unresectable HCC to explore the optimal strategy for this triple therapy approach.

## 2 Materials and methods

### 2.1 Patients

**Figure 1:**
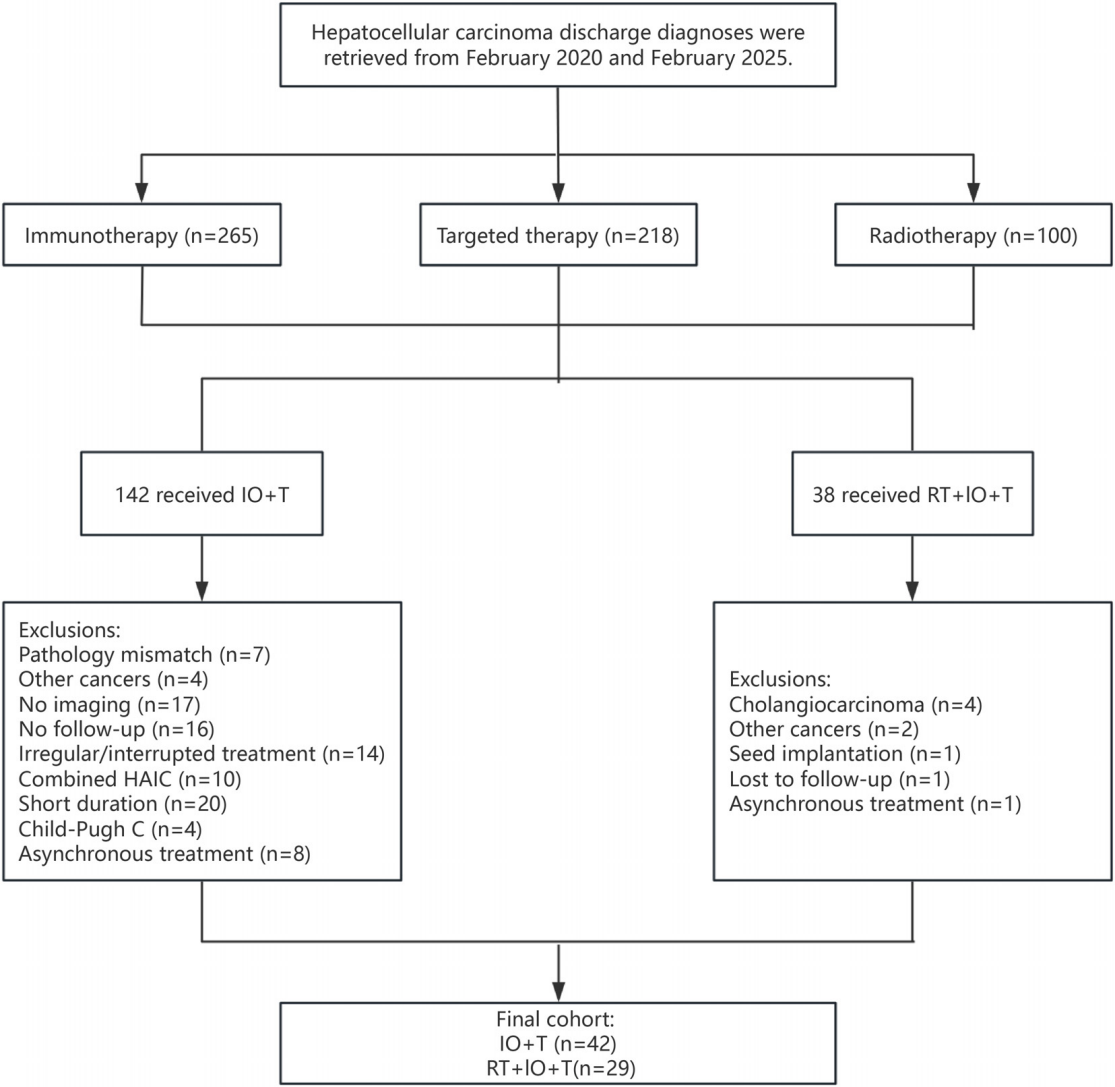
Patient selection flowchart.

This retrospective study reviewed patients with unresectable HCC treated between February 2020 and February 2025 at the Cancer Center of the Sixth Affiliated Hospital of South China University of Technology. Patients received either ICIs with targeted therapy (IO+T) or a triple-modality regimen of RT, ICIs, and targeted therapy (RT+IO+T). All patients were diagnosed with HCC according to clinical or histopathological criteria (3). The inclusion criteria included age >18 years, Child–Pugh class A or B liver function, Eastern Cooperative Oncology Group (ECOG) performance status of 0–2, unresectable HCC determined by surgical assessment or patient refusal of surgery, and at least one measurable intrahepatic lesion according to the modified Response Evaluation Criteria in Solid Tumors mRECIST. The exclusion criteria were other malignancies; other concurrent locoregional therapies, such as transarterial chemoembolization (TACE), hepatic arterial infusion chemotherapy (HAIC), radiofrequency ablation (RFA), or surgical resection, during the study period; incomplete medical records, Child–Pugh class C liver function, or lack of follow-up data (**Figure 1**).

This study was approved by the Ethics Committee of the Sixth Affiliated Hospital of South China University of Technology (Approval No. 2022242) and registered in the Medical Research Information Registration System of the National Health Insurance Information platform (https://www.medicalresearch.org.cn/) (Registration Number: MR-44-23-028102). Given the retrospective nature of this study, the requirement for informed consent was waived in accordance with institutional guidelines. All procedures adhered to the ethical principles of the Declaration of Helsinki (as revised in 2013), ensuring the protection of patient privacy and the confidentiality of medical data.

### 2.2 Treatments

The study participants were administered either IO+T or a RT+IO+T triple-modality regimen. The ICIs included camrelizumab (200 mg), tislelizumab (200 mg), sintilimab (200 mg), or atezolizumab (1200 mg), administered intravenously every three weeks. The targeted agents included lenvatinib, anlotinib, donafenib, bevacizumab, regorafenib, or apatinib, selected at the treating physician’s discretion based on disease characteristics and patient tolerance.

In the RT+IO+T group, RT was delivered using a 6-MV linear accelerator (Varian) via stereotactic body RT (SBRT; 36–60 Gy in 3–10 fractions) or IMRT (IMRT; 50–60 Gy in 20–30 fractions), depending on the tumor location, size, and hepatic reserve. Gross tumor volume (GTV) was defined using contrast-enhanced computed tomography (CT) and, when available, magnetic resonance imaging (MRI). A 5-mm margin was added to the GTV to generate the planning target volume (PTV), accounting for setup variability and respiratory motion. RT was generally initiated 2–3 weeks after the start of immunotherapy and targeted therapy.

The biologically effective dose (BED) was calculated using the linear-quadratic model according to the following equation: BED = nd × [1 + d/(α/β)], where n is the number of fractions, d is the dose per fraction (Gy), and an α/β ratio of 10 Gy was used for tumor tissues (14).

For patients with Barcelona Clinic Liver Cancer (BCLC) stage C disease, the RT primarily targeted intrahepatic tumors. In patients with extrahepatic metastases, radiation was selectively applied to symptomatic or clinically significant lesions, guided by tumor burden, anatomical accessibility, and palliative intent. For example, retroperitoneal lymph node metastases were treated concurrently in select patients, while lesions in the lung or spine were typically managed with sequential RT. These decisions were made to enhance local control, alleviate symptoms, and mitigate the risk of disease progression.

The dose constraints for organs-at-risk (OARs) adhered to standard recommendations (Quantitative Analyses of Normal Tissue Effects in the Clinic/Radiation Therapy Oncology Group [QUANTEC/RTOG[), including a mean liver dose <28 Gy, liver V30 <60%, gastrointestinal Dmax <54 Gy, spinal cord Dmax <45 Gy, and kidney mean dose <18 Gy or V20 <30%, depending on proximity and function. RT plans were individualized to balance optimal tumor coverage with toxicity mitigation, especially in patients with cirrhosis. All patients undergoing RT were monitored closely for treatment-related adverse events, and dose adjustments were made when necessary to preserve safety.

### 2.3 Follow-up

Treatment safety was continuously monitored, and tumor response was evaluated using the mRECIST every two treatment cycles or earlier if clinically warranted (15). The objective response rate (ORR) was defined as the proportion of patients achieving either a complete response (CR) or partial response (PR), while the disease control rate (DCR) encompassed CR, PR, and stable disease (SD). OS was calculated from the initiation of immunotherapy combined with target agents to the date of death or the latest available follow-up. Progression-free survival (PFS) was defined as the time from treatment onset to radiologically confirmed progression or last documented follow-up. The most recent follow-up date was determined by the latest patient contact, including radiologic exams, outpatient notes, telephone communication, or death data sourced from the Guangdong Provincial Medical Death Registry System. Adverse events were graded according to the Common Terminology Criteria for Adverse Events (CTCAE) v4.0, with grade 3 or 4 adverse events classified as severe.

### 2.4 Statistical analysis

All statistical analyses were conducted using R software and IBM SPSS Statistics for Windows, version 26.0 (IBM Corp., Armonk, NY, USA). Continuous variables are summarized as medians with interquartile ranges, whereas categorical variables are reported as counts and percentages. Between-group comparisons were performed using the Mann–Whitney U test and the chi-square test for continuous and categorical data, respectively. Survival outcomes, including OS and PFS, were estimated using the Kaplan–Meier method and compared between groups using the log-rank test. Associations between clinical variables and survival outcomes were further explored through univariate and multivariate analyses using Cox proportional hazard ratio (HR) models. Subgroup analyses were also carried out where appropriate. Two-sided p<0.05 was considered statistically significant.

## 3 Results

### 3.1 Patient baseline characteristics

The analysis included a total of 71 patients with unresectable HCC, with the IO+T and RT+IO+T groups comprising 42 and 29 patients, respectively (**Table 1**). The baseline characteristics were generally comparable between the two groups, with no significant differences in age, sex, Child–Pugh score, alpha-fetoprotein (AFP) levels, maximum tumor diameter, or presence of extrahepatic metastasis. However, the RT+IO+T group had a significantly higher proportion of patients with advanced disease (BCLC stage C: 96.6% vs. 71.4%, p=0.007) and a trend toward more patients showing portal vein invasion (69.0% vs. 47.6%, p=0.075). Although the RT+l0+T had a higher proportion of patients with BCLC stage C disease (96.6% vs.71.4%), they paradoxically exhibited better performance status (ECOG 0: 31.0% vs.11.9%, p=0.046) and were more often treated in the recurrent setting (i.e., second-line or later systemic therapy: 65.5% vs. 23.8%, p<0.001). This apparent discrepancy may be attributed to disease heterogeneity within BCLC stage C. Specifically, patients with macrovascular invasion (e.g., portal vein tumor thrombus [PVTT]) may retain preserved liver function and performance status compared with patients with diffuse intrahepatic tumors or extrahepatic spread, who often present with more severe clinical symptoms.

**Table 1:** Baseline characteristics of the treatment groups.

Variables	Total (n = 71)	IO+T (n = 42)	RT+IO+T (n = 29)	*p*
Age (years), n (%)				*0.886*
<60	35 (49.3)	21 (50)	14 (48.3)	
≥60	36 (50.7)	21 (50)	15 (51.7)	
Gender, n (%)				*0.209*
Female	12 (16.9)	5 (11.9)	7 (24.1)	
Male	59 (83.1)	37 (88.1)	22 (75.9)	
BCLC, n (%)				*0.007*
B	13 (18.3)	12 (28.6)	1 (3.4)	
C	58 (81.7)	30 (71.4)	28 (96.6)	
Child–Pugh score, n (%)				*0.297*
A	57 (80.3)	32 (76.2)	25 (86.2)	
B	14 (19.7)	10 (23.8)	4 (13.8)	
ECOG PS score, n (%)				*0.046*
<1	14 (19.7)	5 (11.9)	9 (31)	
≥1	57 (80.3)	37 (88.1)	20 (69)	
Baseline AFP (ng/mL), n (%)				0.195
<400	40 (56.3)	21 (50)	19 (65.5)	
≥400~1000	31 (43.7)	21 (50)	10 (34.5)	
Maximum tumor diameter (cm), n (%)				*0.375*
<10	55 (77.5)	31 (73.8)	24 (82.8)	
≥10	16 (22.5)	11 (26.2)	5 (17.2)	
Extra Hepatic Metastasis, n (%)				*0.756*
No	45 (63.4)	26 (61.9)	19 (65.5)	
Yes	26 (36.6)	16 (38.1)	10 (34.5)	
Portal vein invasion, n (%)				*0.075*
No	31 (43.7)	22 (52.4)	9 (31)	
Yes	40 (56.3)	20 (47.6)	20 (69)	
Treatment.line, n (%)				< 0.001
First-line	42 (59.2)	32 (76.2)	10 (34.5)	
Second-line and beyond	29 (40.8)	10 (23.8)	19 (65.5)	
Previous Locoregional Therapy, n (%)				0.149
No	41 (57.7)	27 (64.3)	14 (48.3)	
TACE	20 (28.2)	8 (19)	12 (41.4)	
HAIC	10 (14.1)	7 (16.7)	3 (10.3)	

BCLC, Barcelona Clinic Liver Cancer stage; ECOG PS, Eastern Cooperative Oncology Group Performance Status; AFP, alpha-fetoprotein; PFS, progression-free survival; OS, overall survival; IO, immuno-oncology therapy; T, targeted therapy; RT, radiotherapy; TACE, transarterial chemoembolization; HAIC, hepatic arterial infusion chemotherapy.

Collectively, while most baseline characteristics were well balanced, the RT+IO+T cohort presented with both more aggressive disease features and better functional status, which could influence treatment selection and clinical outcomes. RT was administered based on anatomical site and clinical indications. The detailed parameters of RT administered to the combination therapy cohort are presented in **Table 2**. Among 29 patients receiving RT, intrahepatic lesions (51.4%) and PVTT/hepatic vein tumor thrombus (HVTT) (31.4%) were the most common targets. Of the seven patients with extrahepatic metastases, six received RT targeting metastatic sites. Retroperitoneal lymph nodes (8.6%) and lung (2.9%) were treated concurrently, while spinal bone lesions (5.7%) received sequential RT. IMRT and SBRT were employed in 69.0% and 31.0% of patients, respectively. For hepatic lesions, the median total dose was 50.0 Gy (range, 36.0–60.0 Gy) over a median of 20 fractions (range, 6–30). The median BED_10_ was 60.0 Gy (range, 43.2–96.0 Gy). Data on RT parameters were limited to liver-directed treatments and excluded extracranial metastatic sites.

**Table 2:** Details of the radiation therapy parameters used in this study.

Radiation therapy parameters	Total (n = 29)
Radiation.therapy.site, n (%)	
PVTT/HVTT	11 (31.4)
Liver lesions	18 (51.4)
Lung	1 (2.9)
Lymph nodes	3 (8.6)
Bone	2 (5.7)
Radiation therapy technique, no. (%)	
IMRT	20 (69.0)
SBRT	9 (31.0)
Total prescribed dose (Gy), Median (range)	50.0 (36.0, 60.0)
Radiation therapy fraction, Median (range)	20.0 (6.0, 30.0)
BED10(Gy), Median (range)	60.0 (43.2, 96)
Radiation.therapy.site, n (%)	
PVTT/HVTT	11 (31.4)
Liver lesions	18 (51.4)

PVTT/HVTT, portal/hepatic vein tumor thrombosis.

### 3.2 Short-term efficacy

The combination therapy cohort (RT+IO+T) demonstrated significantly enhanced short-term efficacy compared with the IO+T group at 3 months (**Table 3**). The ORR was double in the RT+IO+T arm compared with the IO+T arm (69.0% vs. 35.7%, p=0.006), with PR constituting most of the achieved responses (69.0% vs. 35.7%), no CR were observed. The DCR was also markedly higher in the combination group (89.7% vs. 57.1%, p=0.003), paralleled by a substantial reduction in disease progression (10.3% vs. 42.9%, p=0.003). Finally, the SD rates remained comparable between cohorts (20.7% vs. 21.4%, p=0.940). These findings indicate synergistic antitumor activity when RT is integrated into therapeutic regimens.

**Table 3:** Comparisons of short-term efficacy at 3 months between the treatment cohorts.

Response category	IO+T (n = 42)	RT+IO+T (n = 29)	*P*-value
PR, n (%)	15 (35.7)	20 (69)	0.006
SD, n (%)	9 (21.4)	6 (20.7)	0.94
PD, n (%)	18 (42.9)	3 (10.3)	0.003
ORR, n (%)	15 (35.7)	20 (69)	0.006
DCR, n (%)	24 (57.1)	26 (89.7)	0.003

IO, immuno-oncology therapy; T, targeted therapy; RT, radiotherapy; ORR, objective response rate (PR+CR); DCR, disease control rate (PR+CR+SD); PR, partial response; SD, stable disease; PD, progressive disease.

### 3.3 Safety

Treatment-related adverse events occurred commonly in both groups (**Table 4**), with comparable frequencies of low-grade symptoms including anorexia (58.6% vs. 54.8%, p=0.747), fatigue (51.7% vs. 45.2%, p=0.591), and mild gastrointestinal events including nausea, vomiting, and fever (all p>0.2). These symptoms were generally well tolerated and manageable. However, patients in the RT+IO+T group experienced significantly higher rates of hematologic and hepatic toxicities, including lymphopenia (96.6% vs. 52.4%, p<0.001), thrombocytopenia (75.9% vs. 31.0%, p<0.001), and leukopenia (62.1% vs. 19.0%, p<0.001), among which grade 3–4 lymphopenia (62.1% vs. 19.0%, p<0.001) and thrombocytopenia (24.1% vs. 2.4%, p=0.007) represented key dose-limiting toxicities. Additionally, hepatic enzyme elevations were more common in the RT+IO+T group, with AST increased in 75.9% of patients compared with 35.7% of patients in the IO+T group (p<0.001) and ALT in 51.7% vs. 19.0% of patients (p=0.004). Although gastrointestinal ulceration was observed more frequently in the RT+IO+T group (20.7% vs. 4.8%, p=0.056), this difference was not statistically significant. Despite the higher incidence of select grade ≥3 adverse events, all toxicities were manageable with standard supportive care, with no treatment-related mortality. These findings suggest that while the RT+IO+T regimen is associated with an increased hematologic and hepatic toxicity profile, it remains clinically tolerable and feasible in patients with advanced HCC.

**Table 4:** Treatment-related adverse events.

Adverse event	IO+T (n = 42)		RT+IO+T (n = 29)		*P-value*	
	Any grade adverse event	Grade 3–4 adverse events	Any grade adverse event	Grade 3–4 adverse events	Any grade adverse event	Grade 3–4 adverse events
All Adverse Events, n (%)	40 (95.2)	12 (28.6)	29 (100)	18 (62.1)	0.51	0.005
Anorexia, n (%)	23 (54.8)	0 (0)	17 (58.6)	0 (0)	0.747	–
Nausea, n (%)	4 (9.5)	0 (0)	6 (20.7)	0 (0)	0.298	–
Vomiting, n (%)	1 (2.4)	0 (0)	1 (3.4)	0 (0)	1	–
Fever, n (%)	3 (7.1)	0 (0)	6 (20.7)	0 (0)	0.146	–
Fatigue, n (%)	19 (45.2)	0 (0)	15 (51.7)	0 (0)	0.591	–
Alanine aminotransferase elevation, n (%)	8 (19)	0 (0)	15 (51.7)	0 (0)	0.004	–
Aspartate aminotransferase elevation, n (%)	15 (35.7)	1 (2.4)	22 (75.9)	2 (6.9)	< 0.001	0.563
Thrombocytopenia, n (%)	13 (31)	1 (2.4)	22 (75.9)	7 (24.1)	< 0.001	0.007
Leukopenia, n (%)	8 (19)	0 (0)	18 (62.1)	2 (6.9)	< 0.001	0.163
Anemia, n (%)	9 (21.4)	3 (7.1)	12 (41.4)	6 (20.7)	0.07	0.146
Hypertension, n (%)	1 (2.4)	0 (0)	2 (6.9)	0 (0)	0.563	–
Infection, n (%)	8 (19)	0 (0)	5 (17.2)	0 (0)	0.847	–
Hypothyroidism, n (%)	9 (21.4)	0 (0)	4 (13.8)	0 (0)	0.414	–
Creatinine elevation, n (%)	3 (7.1)	0 (0)	3 (10.3)	0 (0)	0.683	–
Lymphopenia, n (%)	22 (52.4)	8 (19)	28 (96.6)	18 (62.1)	< 0.001	< 0.001
Gastrointestinal ulcer, n (%)	2 (4.8)	0 (0)	6 (20.7)	0 (0)	0.056	–
Esophageal and gastric fundus variceal bleeding, n (%)	3 (7.1)	0 (0)	4 (13.8)	0 (0)	0.433	–
Rash, n (%)	9 (21.4)	2 (4.8)	4 (13.8)		0.414	0.51

IO, immuno-oncology therapy; T, targeted therapy; RT, radiotherapy.

### 3.4 Cox regression analysis

Multivariate Cox regression analysis revealed that the RT+IO+T regimen was independently associated with improved survival outcomes, reinforcing the clinical benefit of adding RT to IO+T. Specifically, RT+IO+T significantly prolonged PFS (HR=0.39, 95% confidence interval [CI]: 0.21–0.73, p=0.003) and OS (HR=0.44, 95% CI: 0.19–0.98, p=0.045). Among clinical variables, BCLC stage C was an independent risk factor for worse OS (HR=3.85, 95% CI: 1.40–10.56, p=0.009) (**Table 5**). While ECOG performance status, tumor diameter, and extrahepatic metastasis were associated with poor prognosis in univariate analysis, these associations did not remain significant after multivariate adjustment. Other factors, including age, sex, hepatitis B virus (HBV) infection status, and prior locoregional therapy, did not significantly correlate with PFS or OS. Collectively, these results highlighted the added therapeutic value of RT in the systemic management of advanced HCC.

**Table 5:** Univariate and multivariate analysis of progression-free survival and overall survival.

Item	PFS				OS			
	Univariate analysis		Multivariate analysis		Univariate analysis		Multivariate analysis	
	HR (95%CI)	*P* value	HR (95%CI)	*P* value	HR (95%CI)	*P*	HR (95%CI)	*P* value
Age: <60 vs ≥60	0.62 (0.38,1.01)	0.055			0.61 (0.33,1.1)	0.101		
Gender: Male vs Female	1.19 (0.61,2.29)	0.606			1.1 (0.49,2.48)	0.814		
HBV: Yes vs No	1.22 (0.66,2.24)	0.519			0.96 (0.47,1.95)	0.901		
ChildPugh: B vs A	0.98 (0.53,1.81)	0.943			1.19 (0.52,2.7)	0.682		
BCLC: C vs B	1.39 (0.72,2.67)	0.308			2.38 (0.99,5.71)	0.032	3.85 (1.4~10.56)	0.009
ECOG PS score: <1 vs ≥1	1.89 (0.97,3.66)	0.045	1.28 (0.62~2.62)	0.505	3.07 (1.19,7.92)	0.008	2.35 (0.86~6.45)	0.097
Maximum tumor diameter(cm): <10 vs ≥10	1.78 (0.98,3.21)	0.07			2.1 (1.08,4.08)	0.038	1.58 (0.77~3.23)	0.214
Extra HepaticMetastasis: Yes vs No	1.67 (0.98,2.84)	0.063			1.97 (1.05,3.69)	0.038	0.96 (0.48~1.94)	0.92
Portal vein invasion: Yes vs No	1.15 (0.7,1.89)	0.589			1.38 (0.75,2.53)	0.291		
Group: RT vs NRT	0.35 (0.21,0.6)	< 0.001	0.39 (0.21~0.73)	0.003	0.43 (0.23,0.82)	0.008	0.44 (0.19~0.98)	0.045
Treatment stage: recurrent vs naive	0.57 (0.34,0.96)	0.03	0.72 (0.42~1.25)	0.246	0.51 (0.26,0.99)	0.039	0.69 (0.34~1.4)	0.306
AFP((ng/mL): <400 vs ≥400	1.4 (0.85,2.3)	0.184			1.3 (0.72,2.34)	0.379		
Previous Locoregional Therapy: Yes vs No	0.95 (0.58,1.57)	0.852			0.71 (0.37,1.33)	0.273		

HR, hazard ratio; CI, confidence interval; PFS, progression-free survival; OS, overall survival; HBV, hepatitis B virus; BCLC, Barcelona Clinic Liver Cancer; ECOG PS, Eastern Cooperative Oncology Group performance status; RT, radiotherapy; NRT, no RT; AFP, alpha-fetoprotein.

### 3.5 Subgroup analysis

**Figure 2:**
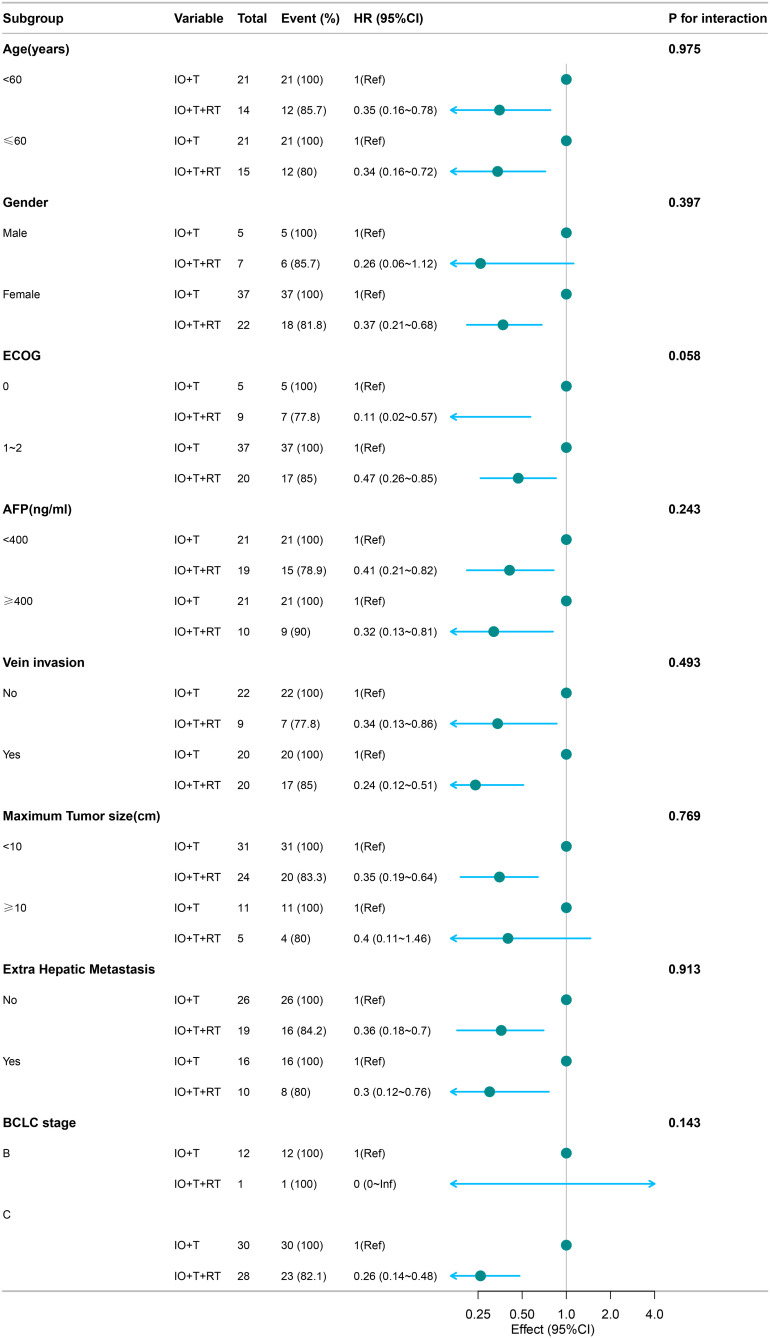
Subgroup analysis of PFS in patients receiving RT+IO+T versus IO+T. The RT+IO+T regimen showed consistently improved PFS across all strata, including both age groups (<60 and ≥60 years), AFP levels, tumor size, portal vein invasion status, and presence of extrahepatic metastasis. No statistically significant interaction was observed across subgroups (all p-interaction >0.05). PFS, progression-free survival; RT, radiotherapy; IO, immunotherapy; T, targeted therapy; AFP, alpha-fetoprotein.

**Figure 3:**
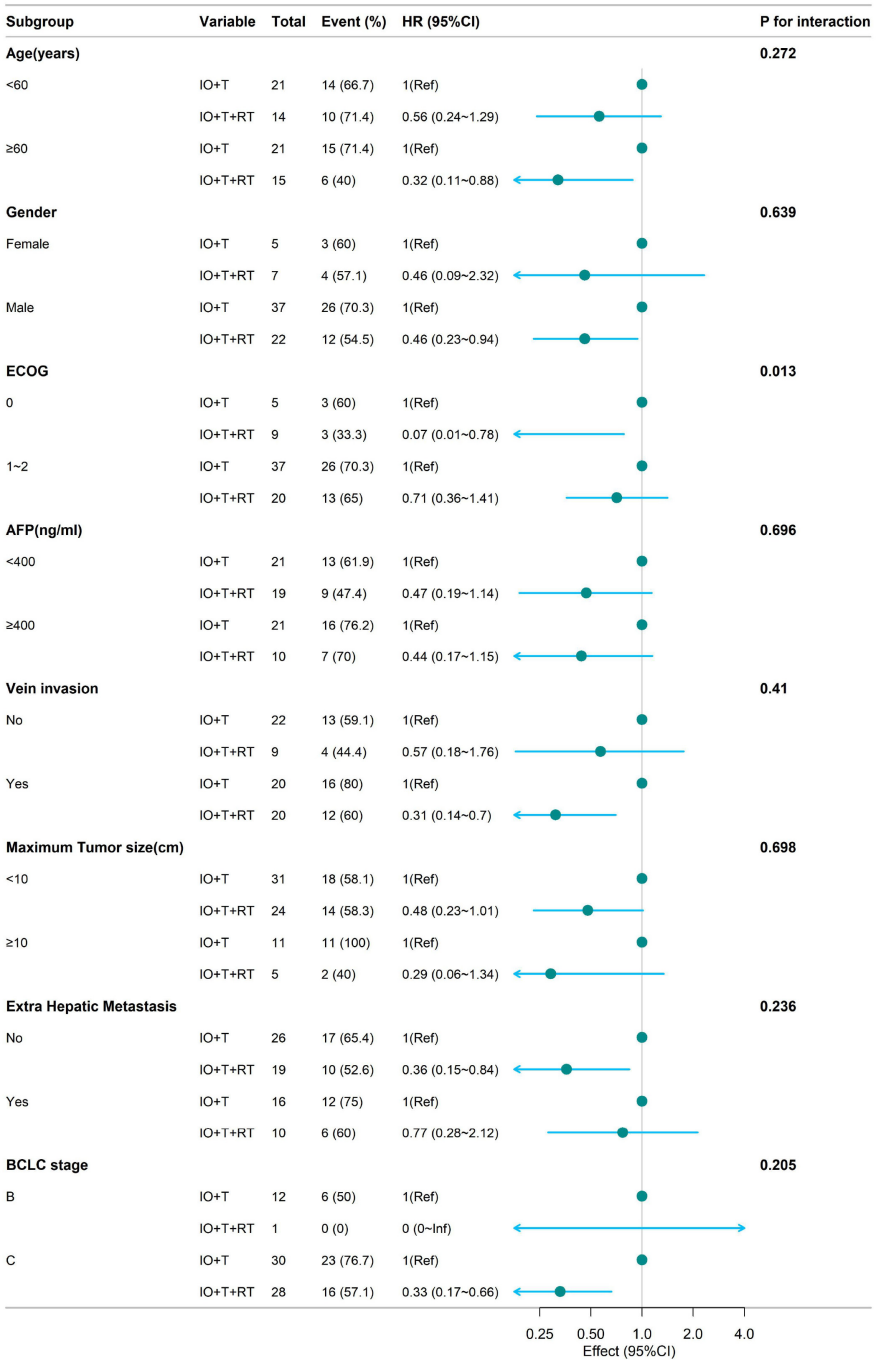
Subgroup analysis of OS in patients receiving RT+IO+T versus IO+T. The RT+IO+T regimen demonstrated a consistent survival benefit across age groups (<60 years: HR = 0.56; ≥60 years: HR = 0.32), sex, AFP levels, tumor size, portal vein invasion, and presence of extrahepatic metastasis. OS, overall survival; RT, radiotherapy; IO, immunotherapy; T, targeted therapy; HR, hazard ratio; AFP, alpha-fetoprotein.

Subgroup analyses for PFS (**Figure 2**) and OS (**Figure 3**) consistently demonstrated the survival benefit of the RT+IO+T regimen across various patient subgroups.

RT+IO+T significantly improved PFS in both age groups (<60 years: HR=0.35, 95% CI: 0.16–0.78; ≥60 years: HR=0.34, 95% CI: 0.16–0.72) without significant interaction by age (p=0.975). Pronounced benefits were also observed in patients with ECOG 0 (HR=0.11, 95% CI: 0.02–0.57) and ECOG 1–2 (HR=0.47, 95% CI: 0.26–0.85), with an interaction trend (p=0.058). The advantage remained consistent regardless of AFP level, portal vein invasion, tumor diameter, or extrahepatic metastasis (**Figure 2**).

Analysis of OS (**Figure 3**) demonstrated a survival benefit of RT+IO+T regardless of age (<60 years: HR=0.56, 95% CI: 0.24–1.29; ≥60 years: HR=0.32, 95% CI: 0.11–0.88), with no significant interaction (p=0.272). Marked benefits were observed in patients with ECOG 0 (HR=0.07, 95% CI: 0.01–0.78), as well as those with ECOG 1–2 (HR=0.71, 95% CI: 0.36–1.41), with a significant interaction (p=0.013). RT+IO+T improved OS in patients with or without portal vein invasion, those with tumors <10 cm, and those with or without extrahepatic metastasis.

The subgroup of patients with BCLC stage C disease who were administered RT+IO+T showed significantly improved PFS (HR=0.33, 95% CI: 0.17–0.66) compared with the IO+T group. Similarly, the RT+IO+T group also showed improved OS in patients with BCLC stage C disease (HR=0.33, 95% CI: 0.17–0.66). In contrast, among patients with BCLC stage B disease, only one patient received RT+IO+T, limiting statistical interpretation. This lack of events in the RT+IO+T subgroup resulted in an HR value of 0 (0–Inf) for OS and precluded reliable comparative analysis. These results highlight the consistent survival benefit of RT+IO+T, especially in patients with better performance status.

### 3.6 Survival analysis

**Figure 4:**
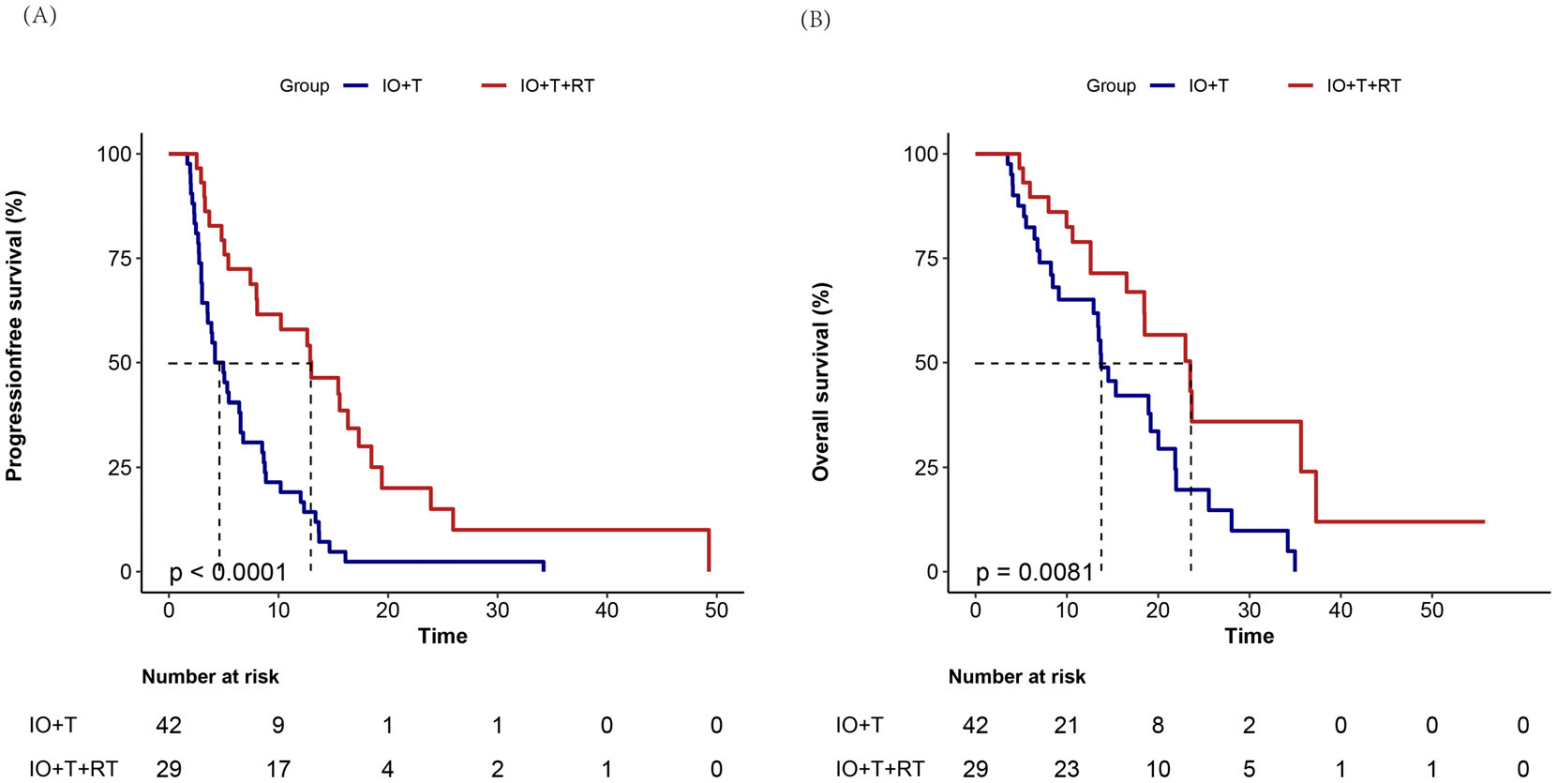
Kaplan–Meier survival curves comparing treatment outcomes between RT+IO+T and IO+T in hepatocellular carcinoma. Compared with the IO+T group, the RT+IO+T group shows superior **(A)** significantly improved progression-free survival (median 12.6 months [95% CI: 5.4–17.3] vs. 4.6 months [95% CI: 2.8–8.7]; p<0.001) and **(B)** OS (median 17.8 months [95% CI: 11.9-23.1] vs. 10.9 months [95% CI: 6.1–17.4]; p=0.009). RT, radiotherapy; IO, immunotherapy; T, targeted therapy; HCC, hepatocellular carcinoma; PFS, progression-free survival; CI, confidence interval; OS, overall survival.

The survival data was censored on April 30, 2025. Kaplan–Meier survival analysis demonstrated a significant survival benefit for both PFS and OS in the RT+IO+T group compared with the IO+T group (**Figure 4**). Specifically, the median PFS in the RT+IO+T group (12.6 months, range: 5.4–17.3 months), significantly longer than that in the IO+T group (4.6 months, range: 2.8–8.7 months) in the IO+T group (p<0.001). The 6-and 12-month PFS values were better in the RT+IO+T group (72.4% vs. 40.5%, 51.7% vs. 19%, respectively, p<0.001).

Similarly, the median OS in the RT+IO+T group was 17.8 months (range: 11.9–23.1 months), compared with 10.9 months (range: 6.1–17.4 months) in the IO+T group (p=0.009). The 6-, 12-, and 24-month OS values were also better in the RT+IO+T group (89.7% vs. 76.2%, 72.48% vs. 50%, and 17.2% vs. 9.5%, respectively, all p>0.05). Notably, the median OS in the RT+RO+T group was 17.8 months, with nine patients still under follow-up. These findings indicate that the addition of RT to the standard IO+T regimen substantially prolonged disease control and OS in patients with unresectable HCC.

**Figure 5:**
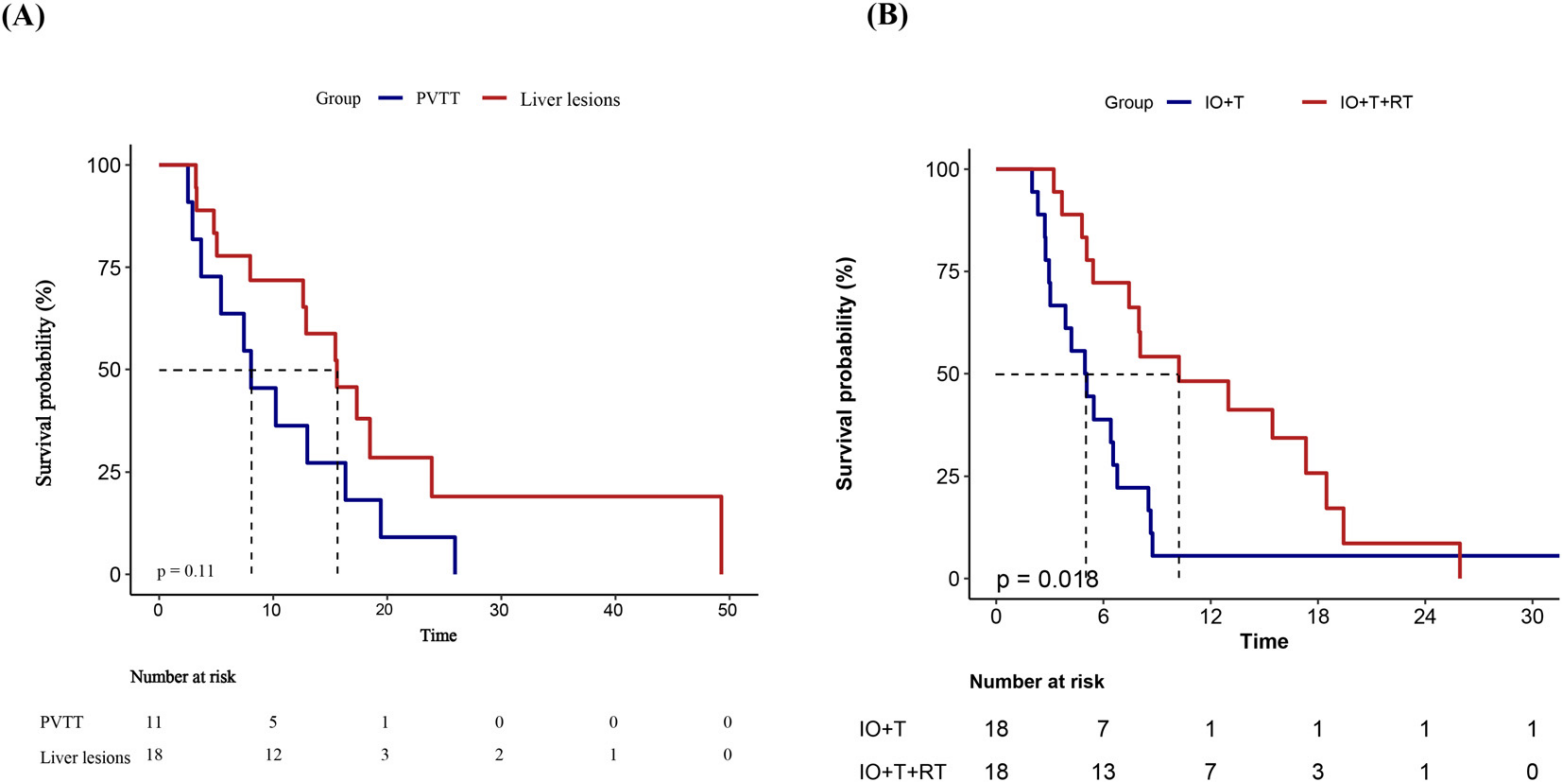
Kaplan–Meier survival curves of PFS in different subgroups.**(A)** PFS comparison between patients receiving RT targeting PVTT versus intrahepatic liver lesions in the RT+IO+T group. Although patients with liver lesion irradiation demonstrated a longer median PFS, the difference was not statistically significant (p = 0.11). **(B)** PFS comparison after 1:1 PSM between the IO+T and RT+IO+T groups. Patients receiving the RT+IO+T regimen showed a significantly prolonged PFS compared with those receiving IO+T alone (p=0.018). PFS, progression-free survival; RT, radiotherapy; PVTT, portal vein tumor thrombus; IO, immunotherapy; T, targeted therapy; PSM, propensity score matching.

Subgroup analysis stratified by RT target site to investigate the potential effects of treatment location on the survival benefit of RT showed a numerically longer PFS among patients receiving liver-directed RT compared with those treated for PVTT (median PFS: 11.4 vs. 7.4 months), although the difference was not statistically significant (p=0.11) (**Figure 5A**). This trend suggests that RT targeting intrahepatic lesions may provide improved local disease control and better synergize with systemic therapies. The absence of statistical significance in the present study may be attributable to limited sample size; therefore, these findings warrant further validation in larger studies.

### 3.7 PSM results

To reduce potential confounding, PSM was performed based on variables associated with PFS in the univariate analysis (p<0.1), including age, ECOG performance status, maximum tumor diameter, extrahepatic metastasis, and treatment line. A 1:1 nearest-neighbor matching algorithm without replacement was applied using a caliper width of 0.2, yielding 18 matched pairs. The baseline characteristics after matching are summarized in **Supplementary Table 1**. Kaplan–Meier analysis of the matched cohort demonstrated a significant improvement in PFS for the RT+IO+T group compared with the IO+T group (median PFS: 9.1 vs. 5.0 months; p=0.018, **Figure 5B**).

Furthermore, after PSM, the RT+IO+T group maintained a statistically significant PFS benefit compared with the IO+T group, with an HR of 0.43 (95% CI: 0.21–0.88, p=0.021) (**Table 6**). These consistent findings suggest that the addition of RT to immunotherapy and targeted therapy significantly prolongs PFS, even after adjusting for baseline differences. These PSM results strengthened the internal validity of the observed treatment effect and supported the robustness of the survival advantage conferred by the triple-modality regimen.

**Table 6:** Propensity score-matched analysis comparing progression-free survival between IO+T and RT+IO+T groups.

Group	Matched patients (n/N)	HR (95%CI)	P value
IO+T	18/42	1 (Reference)	0.021
RT+IO+T	18/29	0.43 (0.21~0.88)	

IO+T, immunotherapy plus targeted therapy; RT+IO+T, RT combined with immunotherapy and targeted therapy; HR, hazard ratio; CI, confidence interval.

## 4 Discussion

The results of this study offer novel evidence of the significant clinical benefits of the addition of RT to IO+T regimens in patients with unresectable HCC. Compared with the IO+T regimen alone, RT+IO+T provided a markedly improved median PFS of 12.6 months vs. 4.6 months (p<0.001), and a median OS of 17.8 months vs. 10.9 months (p=0.009). These survival benefits remained robust in PSM analyses of 18 patient pairs matched for key clinical covariates. RT+IO+T also demonstrated superior short-term efficacy, with higher ORR (69.0% vs. 35.7%, p=0.006) and DCR (89.7% vs. 57.1%, p=0.003). The safety profile was generally acceptable, although higher incidences of grade ≥3 hepatotoxicity and lymphopenia were observed in the RT+IO+T group. However, no treatment-related deaths occurred, and most adverse events were manageable with supportive care. These findings underscore the therapeutic potential and tolerability of integrating RT into the standard IO+T treatment paradigm for advanced HCC.

Previous small-scale studies have suggested that triple-modality approaches incorporating the addition of SBRT or proton beam therapy to atezolizumab and bevacizumab may still confer survival benefits even in patients with highly advanced HCC (16, 17). Previous studies have explored the combination of immunotherapy, targeted therapy, and RT in unresectable HCC, focusing primarily on patients with BCLC stage C and PVTT (4, 14, 16, 18–20). These retrospective, single-center studies assessed efficacy endpoints including PFS, OS, and ORR, with some incorporating PSM and reporting BED parameters (4, 14, 16). In contrast, the present study included a more heterogeneous real-world population treated with diverse ICIs and targeted agents, reported detailed RT parameters and target sites (e.g., PVTT, intrahepatic, and extrahepatic lesions), and conducted site-specific and multi-method PSM analyses, thereby enhancing the robustness and clinical relevance of the findings. A recent matched cohort study in advanced HCC showed that adding RT to ICIs and targeted therapy significantly improved outcomes, with a median PFS of 8.3 vs. 4.2 months, ORR of 75.9% vs. 24.1%, and DCR of 100% vs. 75.9% compared with the non-RT group. The median OS was not reached in the RT group but was significantly prolonged (p=0.002) (21). In comparison, our study demonstrated even longer PFS (12.6 months), high ORR (69.0%), and a median OS of 17.8 months, despite a higher proportion of patients with advanced-stage disease. These results reinforce the added value of RT in combination with immunotherapy and targeted therapy, supporting its broader integration into treatment strategies for advanced HCC.

Compared with patient populations in other studies evaluating the combination of ICIs and antiangiogenic agents, such as IMbrave150, ORIENT-32, RESCUE, CARES-310, and APOLLO (22–26), the cohort in the present study exhibited a higher prevalence of poor prognostic factors, including greater baseline tumor burden, a higher incidence of PVTT, and more frequent occurrence of distant metastases. These high-risk features may partially explain the relatively shorter PFS of 4.6 months and OS of 10.9 months observed in the IO+T group compared with previous studies. The addition of RT to the IO+T regimen yielded significantly improved outcomes across multiple efficacy parameters. While our cohort achieved a median OS of 17.8 months, comparable to 19.2 months reported for the IMbrave150, it demonstrated superior PFS (12.6 vs. 6.8 months) and remarkable improvements in tumor response rates (ORR 69.0% vs. 27.3%, DCR 89.7% vs. 73.6%) (22). This comprehensive advantage was consistently observed in cross-trial comparisons; compared with ORIENT-32 (PFS 4.6 months, ORR 20.3%, DCR 66.7%), the RT+IO+T regimen in the present study showed a 2.7-fold longer PFS with substantially higher ORR and DCR (23). Compared with the RESCUE trial, which reported a PFS of 5.5–5.7 months (ORR 34.3–46.0%, DCR 77.2–84.5%), we observed superior disease control across all efficacy measures (24). Our results also exceeded the outcomes reported in the CARES-310 trial for all evaluated endpoints (PFS 5.6 months, ORR 25.4%, DCR 78.3%) (25). Finally, compared with the APOLLO outcomes (PFS 6.9 months, ORR 13.6%, DCR 73.8%), the regimen in the present study also demonstrated significant advantages in both survival and response metrics (26).

Compared with other locoregional strategies combined with systemic therapy, the RT+IO+T regimen demonstrated favorable efficacy and safety in patients with advanced HCC. TACE combined with tyrosine kinase inhibitors and ICIs has demonstrated an ORR of 50.9%, PFS of 9.1 months, and OS of 19.1 months (27), while TACE combined with molecular-targeted agents plus ICIs achieved an ORR of 65.1%, PFS of 15.4 months, and OS of 27.2 months in selected high-burden cases (28). Tang et al. reported that HAIC combined with lenvatinib and tislelizumab yielded an ORR of 77.1%, a median PFS of 6.6 months, and an OS of 23.2 months in patients with Vp4 portal vein invasion (29). Li et al. demonstrated that HAIC plus camrelizumab and rivoceranib achieved a median PFS of 10.0 months, OS of 19.6 months, ORR of 55.5%, and DCR of 89.0% in patients with PVTT (30). Compared with these locoregional combinations, the RT+IO+T regimen in the present study showed strong tumor control efficacy. While TACE- or HAIC-based strategies may offer comparable or even higher OS in select populations, RT+IO+T provides a non-invasive and highly tolerable alternative, potentially offering broad applicability and favorable safety profiles for patients with advanced-stage HCC.

Although the RT+IO+T regimen was associated with a higher rate of treatment-related adverse events, including significantly more grade ≥3 toxicities than the IO+T group, these events were generally manageable, and no treatment-related deaths occurred. The most common severe toxicities were thrombocytopenia (24.1% vs. 2.4%, p<0.001) and lymphopenia (62.1% vs. 18.6%, p<0.001), likely related to radiation exposure and cumulative hematologic effects (31, 32). Given that many patients had advanced liver disease and portal hypertension, baseline hypersplenism may have contributed to cytopenias (33). Increased rates of variceal bleeding and gastrointestinal ulcers in the RT+IO+T group highlight the need for pre-treatment endoscopic evaluation in high-risk patients. Compared with TACE- or HAIC-based regimens, RT+IO+T demonstrated a comparable or lower incidence of severe adverse events (29, 34–36) while offering a non-invasive approach. These results support RT+IO+T as a well-tolerated and practical treatment option for patients with advanced HCC.

This notable survival benefit may be attributed to the synergistic and immunomodulatory interactions among RT, targeted therapy, and programmed cell death protein 1/programmed cell death-ligand 1 pathway blockade (13, 30, 37). Targeted agents help normalize the tumor vasculature and improve perfusion, thereby enhancing the delivery and efficacy of both RT and immunotherapy (12, 38, 39). Additionally, RT may trigger systemic immune responses, including the abscopal effect, which is further amplified when combined with ICIs (10, 11). The results of our subgroup analysis stratified by radiotherapy target revealed the association of liver-directed radiotherapy with longer PFS compared to RT targeting PVTT. This disparity may be attributed to enhanced radiosensitization and immune modulation within the hepatic parenchyma. Unlike PVTT lesions, which are often poorly perfused, hypoxic, and exhibit a suppressive immune microenvironment, irradiation of intrahepatic tumors benefits from transient vascular normalization induced by antiangiogenic therapy, thereby improving perfusion and oxygen delivery, enhancing radiosensitivity, and mitigating hypoxia-driven resistance (40–43). Well-oxygenated liver tumors also respond more favorably to radiation due to increased DNA damage and reduced hypoxia-mediated radioresistance (44). Furthermore, hepatic irradiation promotes immunogenic cell death, upregulation of major histocompatibility complex I (MHC-I), and increased infiltration of cytotoxic T lymphocytes. These effects are further amplified when combined with ICIs and targeted therapeutic agents (45, 46). Lastly, the liver’s unique immunologic architecture, with spatial proximity between hepatic dendritic cells and sinusoidal antigen-presenting cells, may facilitate stronger immune priming in response to liver-targeted radiation (41). Collectively, these mechanisms offer a compelling explanation for the superior PFS observed for liver-targeted radiotherapy compared to PVTT-directed treatment. The integration of RT, ICI, and targeted therapy exerts synergistic effects to enhance tumor antigen presentation, improve vascular normalization and oxygenation, and amplify cytotoxic immune responses, thereby overcoming the individual limitations of each modality and maximizing therapeutic efficacy in advanced HCC.

Despite its promising findings, this study has some limitations. First, owing to the single-center retrospective cohort design, methodological constraints are unavoidable, including selection bias, information bias, and residual confounding. Additionally, the patients were not randomized, and the decision to administer RT was based on clinical judgment. Hence, patients in the IO+T group may have been excluded from receiving RT due to contraindications such as tumor location or poor performance status, introducing potential baseline differences that could influence PFS outcomes. Second, although baseline characteristics were generally well balanced between the two groups—except for BCLC stage and ECOG performance status—unmeasured confounding cannot be entirely ruled out. In addition, post-progression treatment strategies, such as the use of second- or third-line therapies, may have differed between groups, potentially impacting OS comparisons. Third, the relatively small sample size, particularly the number of events, limited the statistical power of the multivariable adjustment and subgroup analysis. The wide confidence intervals observed in Cox models further highlight potential instability in the survival estimates, highlighting the need for cautious interpretation. Nevertheless, the observed improvements in key clinical endpoints, including PFS, OS, ORR, and DCR, suggest adding RT to immunotherapy and targeted therapy may provide a meaningful therapeutic advantage. Finally, owing to the retrospective study design, quality-of-life data were not systematically collected, which limited assessment of the patient-reported impact of adding RT. Given the potential for increased toxicity, this remains a relevant concern. These findings highlight the need for prospective, multicenter randomized trials incorporating standardized quality of life measures to validate survival benefits and define the optimal role of RT in advanced HCC combination strategies.

## 5 Conclusion

The incorporation of RT into systemic regimens combining ICIs with targeted therapy showed promising tolerability in patients with unresectable HCC. This multimodal strategy appears to enhance therapeutic efficacy, offering higher objective response rates and clinically meaningful gains in both PFS and OS compared with dual-agent approaches.

## Data Availability

The raw data supporting the conclusions of this article will be made available by the authors, without undue reservation.
