# Supplementary material for: Efficacy and safety of radiotherapy combined with immunotherapy and targeted therapy versus immunotherapy plus targeted therapy alone in unresectable hepatocellular carcinoma: a retrospective study
Source: Front Oncol. 2025 Aug 20;15:1643304. doi: 10.3389/fonc.2025.1643304 (PMC12404930; doi:10.3389/fonc.2025.1643304)
Supplement: Supplementary file 1 [file Table1.docx]

Supplementary Material

# Supplementary Table 1

Baseline Characteristics of Patients After PSM

| Variables | Total (n = 36) | 0 (n = 18) | 1 (n = 18) | *p* |
| --- | --- | --- | --- | --- |
| Age (years), n (%) |  |  |  | 0.738 |
| <60 | 19 (52.8) | 9 (50) | 10 (55.6) |  |
| ≥60 | 17 (47.2) | 9 (50) | 8 (44.4) |  |
| Gender, n (%) |  |  |  | 0.228 |
| Female | 8 (22.2) | 2 (11.1) | 6 (33.3) |  |
| Male | 28 (77.8) | 16 (88.9) | 12 (66.7) |  |
| BCLC, n (%) |  |  |  | 0.104 |
| B | 4 (11.1) | 4 (22.2) | 0 (0) |  |
| C | 32 (88.9) | 14 (77.8) | 18 (100) |  |
| ChildPugh, n (%) |  |  |  | 0.402 |
| A | 29 (80.6) | 13 (72.2) | 16 (88.9) |  |
| B | 7 (19.4) | 5 (27.8) | 2 (11.1) |  |
| ECOG, n (%) |  |  |  | 1 |
| 0 | 6 (16.7) | 3 (16.7) | 3 (16.7) |  |
| 1 | 27 (75.0) | 13 (72.2) | 14 (77.8) |  |
| 2 | 3 ( 8.3) | 2 (11.1) | 1 (5.6) |  |
| AFP(ng/mL), n (%) |  |  |  | 0.494 |
| <400 | 22 (61.1) | 10 (55.6) | 12 (66.7) |  |
| ≥400~1000 | 14 (38.9) | 8 (44.4) | 6 (33.3) |  |
| MaximumTumor size(cm), n (%) |  |  |  | 1 |
| <10 | 31 (86.1) | 16 (88.9) | 15 (83.3) |  |
| ≥10 | 5 (13.9) | 2 (11.1) | 3 (16.7) |  |
| Extra Hepatic Metastasis, n (%) |  |  |  | 0.738 |
| No | 19 (52.8) | 9 (50) | 10 (55.6) |  |
| Yes | 17 (47.2) | 9 (50) | 8 (44.4) |  |
| Portal vein invasion, n (%) |  |  |  | 0.18 |
| No | 16 (44.4) | 10 (55.6) | 6 (33.3) |  |
| Yes | 20 (55.6) | 8 (44.4) | 12 (66.7) |  |
| Previous Locoregional Therapy, n (%) |  |  |  | 0.798 |
| No | 19 (52.8) | 9 (50) | 10 (55.6) |  |
| TACE | 11 (30.6) | 5 (27.8) | 6 (33.3) |  |
| HAIC | 6 (16.7) | 4 (22.2) | 2 (11.1) |  |
| Treatment.line, n (%) |  |  |  | 1 |
| First-line | 18 (50.0) | 9 (50) | 9 (50) |  |
| Second-line and beyond | 18 (50.0) | 9 (50) | 9 (50) |  |

Abbreviations: BCLC, Barcelona Clinic Liver Cancer stage; ECOG , Eastern Cooperative Oncology Group; AFP, alpha-fetoprotein; PFS, progression-free survival; OS, overall survival; IO, immuno-oncology therapy; T, targeted therapy; RT, radiotherapy; TACE, transarterial chemoembolization; HAIC, hepatic arterial infusion chemotherapy.
